# Study protocol of the FIRE-8 (AIO-KRK/YMO-0519) trial: a prospective, randomized, open-label, multicenter phase II trial investigating the efficacy of trifluridine/tipiracil plus panitumumab versus trifluridine/tipiracil plus bevacizumab as first-line treatment in patients with metastatic colorectal cancer

**DOI:** 10.1186/s12885-022-09892-8

**Published:** 2022-07-27

**Authors:** G. Sommerhäuser, A. Kurreck, S. Stintzing, V. Heinemann, L. Fischer von Weikersthal, T. Dechow, F. Kaiser, M. Karthaus, I. Schwaner, M. Fuchs, A. König, C. Roderburg, I. Hoyer, M. Quante, A. Kiani, S. Fruehauf, L. Müller, A. Reinacher-Schick, T. J. Ettrich, A. Stahler, D. P. Modest

**Affiliations:** 1 Department of Hematology, Oncology, and Cancer Immunology (CVK/CCM), Charité – Universitätsmedizin Berlin, corporate member of Freie Universität Berlin and Humboldt-Universität zu Berlin, Berlin, Germany; 2 German Cancer Consortium (DKTK), DKFZ, Heidelberg, Germany; 3 Department of Hematology/Oncology, LMU Klinikum, University of Munich, Comprehensive Cancer Center Munich, Munich, Germany; 4 Gesundheitszentrum St. Marien, Amberg, Germany; 5 Oncological Practice, Ravensburg, Germany; 6 Oncological Practice, Landshut, Germany; 7 Department of Hematology and Oncology, Klinikum Neuperlach/ Klinikum Harlaching, Munich, Germany; 8 Oncological Practice Kurfuerstendamm, Berlin, Germany; 9 Department of Gastroenterology, Hepatology, and Gastrointestinal Oncology, München Klinik Bogenhausen, Munich, Germany; 10 Department of Gastroenterology and Gastrointestinal Oncology Goettingen, University Medical Center Goettingen, Goettingen, Germany; 11 Department of Gastroenterology, Hepatology, and Infectiology, University Medical Center Duesseldorf, Duesseldorf, Germany; 12 Department of Gastroenterology, Hepatology, Endocrinology, and Infectiology, Albert-Ludwigs-Universität Freiburg, Freiburg, Germany; 13 Department of Medicine IV, Klinikum Bayreuth GmbH, Bayreuth, Germany; 14 Department of Hematology, Oncology, and Palliative Care, Klinik Dr. Hancken GmbH, Stade, Germany; 15 Onkologie UnterEms, Leer, Germany; 16 Department of Hematology, Oncology and Palliative Care, Ruhr-University Bochum, Bochum, Germany; 17 Department of Internal Medicine, University Hospital Ulm, Ulm, Germany

**Keywords:** Metastatic Colorectal Cancer, Trifluridine/tipiracil, Bevacizumab, Panitumumab, First-line treatment

## Abstract

**Background:**

Initial systemic therapy for patients with metastatic colorectal cancer (mCRC) is usually based on two- or three-drug chemotherapy regimens with fluoropyrimidine (5-fluorouracil (5-FU) or capecitabine), oxaliplatin and/or irinotecan, combined with either anti-VEGF (bevacizumab) or, for RAS wild-type (WT) tumors, anti-EGFR antibodies (panitumumab or cetuximab). Recommendations for patients who are not eligible for intensive combination therapies are limited and include fluoropyrimidine plus bevacizumab or single agent anti-EGFR antibody treatment. The use of a monochemotherapy concept of trifluridine/ tipiracil in combination with monoclonal antibodies is not approved for first-line therapy, yet. Results from the phase II TASCO trial evaluating trifluridine/ tipiracil plus bevacicumab in first-line treatment of mCRC patients and from the phase I/II APOLLON trial investigating trifluridine/ tipiracil plus panitumumab in pre-treated mCRC patients suggest favourable activity and tolerability of these new therapeutic approaches.

**Methods:**

FIRE-8 (NCT05007132) is a prospective, randomized, open-label, multicenter phase II study which aims to evaluate the efficacy of first-line treatment with trifluridine/tipiracil (35 mg/m^2^ body surface area (BSA), orally twice daily on days 1–5 and 8–12, q28 days) plus either the anti-EGFR antibody panitumumab (6 mg/kg body weight, intravenously on day 1 and 15, q28 days) [arm A] or (as control arm) the anti-VEGF antibody bevacizumab (5 mg/kg body weight, intravenously on day 1 and 15, q28 days) [arm B] in *RAS* WT mCRC patients. The primary objective is to demonstrate an improved objective response rate (ORR) according to RECIST 1.1 from 30% (control arm) to 55% with panitumumab. With a power of 80% and a two-sided significance level of 0.05, 138 evaluable patients are needed. Given an estimated drop-out rate of 10%, 153 patients will be enrolled.

**Discussion:**

To the best of our knowledge, this is the first phase II trial to evaluate the efficacy of trifluridine/tipiracil plus panitumumab in first-line treatment of *RAS* WT mCRC patients. The administration of anti-EGFR antibodies rather than anti-VEGF antibodies in combination with trifluridine/tipiracil may result in an increased initial efficacy.

**Trial registration:**

EU Clinical Trials Register (EudraCT) 2019-004223-20. Registered October 22, 2019, ClinicalTrials.govNCT05007132. Registered on August 12, 2021.

**Supplementary Information:**

The online version contains supplementary material available at 10.1186/s12885-022-09892-8.

## Background

Colorectal cancer (CRC) represents the third most diagnosed cancer and the third most common cause of cancer related mortality worldwide [1]. At the time of initial diagnosis, 20% of patients present metastatic disease with a 5-year survival rate of less than 20% [2]. The optimal combination and sequence of available systemic therapies is largely determined by the need for tumor remission and the patient’s age and health status. However, since more than half of the patients with CRC in the western world are diagnosed beyond the age of 70 years, there is a clinical need for age- and comorbidity-adjusted treatment strategies.

Combinations of cytotoxic agents including fluoropyrimidine (FP), oxaliplatin and/or irinotecan in combination with either anti-VEGF or, for RAS WT tumors, anti-EGFR antibodies represent the current standard of first-line treatment for patients with metastatic colorectal cancer (mCRC) [3]. For patients who are unfit or unwilling to either undergo combination chemotherapy or up-front surgery for metastatic disease, the primary therapeutic aim is to prevent tumor progression and prolong survival with little impairment of quality of life. Evidence from randomized clinical trials on effective and better tolerable treatment alternatives focuses on combinations of FP and bevacizumab. Consistently, these studies report response rates ranging from 19 to 38%, with a median progression-free survival (PFS) of 8 to 9 months, and a median overall survival (OS) of 21 to 22 months [4–7]. Current evidence from randomized clinical trials demonstrates that first-line treatment with FP plus bevacizumab represents a valuable treatment option not only for patients with disseminated metastases and without the need to achieve rapid tumor shrinkage, but also for patients who are not eligible for combination chemotherapy.

By contrast, the administration of FP monochemotherapy in combination with an anti-EGFR antibody is not approved by a phase 3 trial. However, the randomized phase II PANDA study compared folinic acid, 5-flourouracil and oxaliplatin (FOLFOX) plus panitumumab (arm A) and FP monochemotherapy plus panitumumab (arm B) in a RAS WT mCRC first-line treatment setting [8]. The authors of the PANDA study hypothesized that anti-EGFR combination with FP monotherapy instead of doublet chemotherapy might prove similarly efficient in elderly mCRC patients. The primary endpoint PFS was met in both treatment arms (9.6 [95% CI: 8.8–10.9] versus 9.1 [95% CI: 7.7–9.9] months), with a similar objective response rate (ORR), and disease control rate (DCR) suggesting that FP plus panitumumab is a reasonable option in elderly *RAS* WT mCRC patients.

The phase II TASCO trial [9] compared the efficacy and safety of another monochemotherapeutic agent, that has already been approved in third-line treatment of mCRC [10], in combination with bevacizumab to the standard regimen capecitabine plus bevacizumab in untreated mCRC patients. The efficacy was comparable in both treatment arms with trifluridine/tipiracil plus bevacizumab trending towards improved progression-free survival (9.2 [95% CI: 6.0–9.7] versus 7.8 [95% CI: 4.1–9.1] months) [9]. Overall, the treatment was well tolerated without a significant difference in patient reported outcomes compared to the standard treatment arm. Although, the respective phase III trial (SOLSTICE) failed to demonstrate superiority in terms of PFS with trifluridine/tipiracil plus bevacizumab versus capecitabine plus bevacizumab (Hazard ratio (HR) 0.87, *P* = 0.09), the numerical trend in favour of the trifluridine/tipiracil plus bevacizumab arm suggests, that this regimen is a clinically active despite the risk that it will not become a labelled option [11].

While the combination of bevacizumab plus monochemotherapy appears established in first-line therapy of mCRC, this is less evident for EGFR-targeted agents in combination with fluoropyrimidines and derivates. Thus, patients who are ineligible for combination chemotherapy do not benefit from anti-tumor activity of anti-EGFR antibodies. This is particularly unfortunate as selected derive a substantial clinical benefit in terms of response rate and overall survival from these agents [12, 13]. Corresponding to these encouraging data, the phase I/II APOLLON study [14] evaluated trifluridine/ tipiracil plus panitumumab in patients with pre-treated mCRC with *RAS* WT and demonstrated promising activity (ORR: 37% [95% CI: 24.3–51.3]).

Based on the aforementioned data, the use of anti-EGFR antibodies compared to anti-VEGF antibodies may result in a substantial clinical benefit in combination with chemotherapeutic monotherapy such as trifluridine/tipiracil and is worth being evaluated in a randomized clinical trial.

## Objectives and study endpoints

### Objectives

The primary objective of the FIRE-8 trial is to assess the efficacy of trifluridine/tipiracil plus panitumumab compared to trifluridine/tipiracil plus bevacizumab as first-line treatment for patients with mCRC who are unfit for combination chemotherapy (Table 1).

**Table 1:** Objectives and endpoints of the FIRE-8 trial

**1. Objectives**	
**1.1 Primary Objective**	
■ To compare the efficacy of treatment with trifluridine/tipiracil plus panitumumab versus trifluridine/tipiracil plus bevacizumab	
**1.2 Secondary Objectives**	
■ To compare efficacy, safety and patient reported quality of life (QoL) of treatment with trifluridine/tipiracil plus panitumumab versus trifluridine/tipiracil plus bevacizumab	
**1.3 Other Exploratory Objectives**	
■ Further anti-tumor treatment after discontinuation of study treatment	
**1.4 Translational Research Objectives**	
■ Identification and characterization of patient subgroups with greatest or lowest benefit from respective treatment including efficacy and toxicity	
**2. Endpoints**	
**2.1 Primary Endpoints**	
■ Objective response rate (ORR) according to RECIST 1.1 (assessment at the local trial center)	
**2.2 Secondary Endpoints**	
**Efficacy**	
■ Overall survival (OS)	
■ Progression-free survival (PFS)	
■ Objective response rate (ORR) according to RECIST 1.1 (assessment by central review)	
■ Depth of response (DoR) (assessment by central review)	
■ Early tumor shrinkage ([ETS]; assessment by central review)	
**Quality of Life**	
■ QoL as assessed with the QoL questionnaire EQ-5D-5L	
**Safety**	
■ Type, incidence, severity, and causal relationship to investigational medicinal products (IMPs) of non-serious adverse events (AE) and serious adverse events (SAE; severity evaluated according to CTCAE version 5.0)	
**2.3 Other Exploratory Endpoints**	
■ Subsequent anti-tumor treatment lines (monotherapy and combination therapy treatment lines including medicinal products [chemotherapeutics, antibodies and targeted therapy] and investigator reported efficacy of subsequent treatment lines	

### Study endpoints

The primary study endpoint is the objective response rate (ORR) defined as complete and partial remissions according to RECIST 1.1 as assessed by the investigators. Secondary efficacy endpoints include OS, PFS and as assessed by central review ORR, depth of response (DpR) and early tumor shrinkage (ETS). QoL (EQ-5D-5L), safety and tolerability (National Cancer Institute Common Toxicity Criteria for Adverse Events; NCI-CTCAE) are also assessed (Table 1).

## Methods

### Trial design

The FIRE-8 trial is a prospective, randomized, open-label, multicenter phase II study coordinated and sponsored by Charité- Universitaetsmedizin Berlin. The study will be conducted in 40 study sites in Germany with a planned enrolment of 153 patients.

**Fig. 1:**
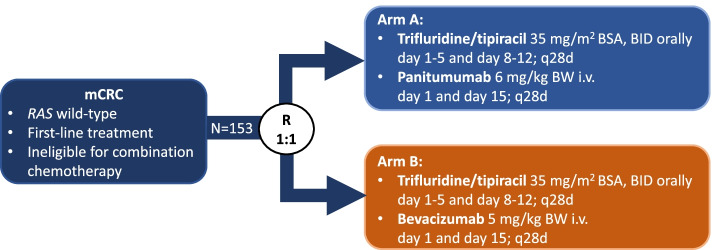
FIRE-8 Study Design. Legend: mCRC, metastatic colorectal cancer; R, randomization; BSA, body surface area; BID, twice daily; BW, body weight. Microsoft PowerPoint version 16.62 was used to generate this figure

Eligible patients with *RAS* WT mCRC are randomly assigned in a 1:1 ratio to either receive trifluridine/tipiracil in combination with panitumumab (Arm A) or trifluridine/tipiracil plus bevacizumab (Arm B) in first-line treatment setting (Fig. 1). Patients will be randomized after verification of eligibility criteria according to a randomization plan generated prior to the clinical trial by the ClinAssess Biometrics Department. Randomization is performed using the following stratification factors: the patient’s performance status (ECOG 0 vs. ECOG 1–2) and the occurence of metastasis (synchronous vs. metachronous). The randomization list (separately for each strata) follows a permuted block design with number of patients equally for both arms in each block. Trial assessments and procedures are outlined in an additional table (Additional file 1).

### Study duration

Patient recruitment started in December 2021. The recruitment period is planned to be completed within 36 months, while the primary endpoint of the study is expected to be evaluated in 2025. Treatment in both study arms will be continued until disease progression according to RECIST 1.1 criteria as assessed by the investigator or until the occurrence of unacceptable toxicity. Follow-up will be performed until the patient’s death, withdrawal from the study or for a minimum of 5 years from time of randomization, whichever occurs first. The last patient visit is scheduled for QIII 2029.

### Trial population

Patients with *RAS* WT mCRC, treated in participating hospitals and oncology practices in Germany, who fulfill all of the following inclusion criteria and have none of the exclusion criteria are eligible for trial participation.

#### Inclusion Criteria

1. Patient’s signed informed consent
2. Patients ≥18 years at the time of signing the informed consent
3. Histologically confirmed adenocarcinoma of the colon or rectum
4. Metastatic colorectal cancer (mCRC) with at least one measurable lesion according to RECIST 1.1 in a computed tomography (CT) or magnetic resonance imaging (MRI) scan performed within 5 weeks prior to randomization
5. Metastases are primarily unresectable or patient is unable/unwilling to undergo surgery
6. RAS WT (KRAS, exons 2, 3, 4 and NRAS, exons 2, 3, 4) mCRC, proven in the primary tumor or metastasis. The RAS mutational status must be determined by means of a validated test method.
7. Patient is not eligible to undergo combination chemotherapy according to investigator’s assessment or unwilling to undergo combination chemotherapy.
8. ECOG performance status 0–2
9. Adequate bone marrow, hepatic and renal organ function, defined by the following laboratory test results:

- Absolute neutrophil count ≥1.5 × 10^9^/L (1500/μL)
- Hemoglobin ≥80 g/L (8 g/dL)
- Platelet count ≥75 × 10^9^/L (75,000/μL) without transfusion
- Total serum bilirubin of ≤1.5 x upper limit of normal (ULN)
- Aspartate aminotransferase (AST/GOT) and alanine aminotransferase (ALT/GPT) ≤ 2.5 × ULN; if liver function abnormalities are due to underlying liver metastasis, AST and ALT ≤5 × ULN
- Calculated glomerular filtration rate (GFR) according to Cockcroft –Gault formula or according to MDRD ≥30 mL/min or serum creatinine ≤1.5 x ULN
- Urine dipstick for proteinuria < 2+ (within 14 days prior to randomization), unless a subsequent 24-hour urine collection demonstrates < 1 g of protein in 24 hours.

10. Patients without anticoagulation need to present with an International Normalized Ratio (INR) < 1.5 x ULN and PTT < 1.5 x ULN. Patients with anticoagulation may be enrolled if the patient receives the medication at a stable dose for at least 2 weeks before randomization and provided that INR and PTT are < 1.5 xULN.
11. For females of childbearing potential (FCBP): negative pregnancy test within 14 days before randomization and agreement to remain abstinent (refrain from heterosexual intercourse) or use contraceptive methods with a failure rate of < 1% per year during the treatment period and for at least 6 months after the last dose of study treatment. A woman is considered to be of childbearing potential if she is postmenarcheal, has not reached a postmenopausal state (≥ 12 continuous months of amenorrhea with no identified cause other than menopause), and has not undergone surgical sterilization (removal of ovaries and/or uterus). Examples of contraceptive methods with a failure rate of < 1% per year include bilateral tubal ligation, male partner’s sterilization, hormonal contraceptives that inhibit ovulation, hormone- releasing intrauterine devices, and copper intrauterine devices. The reliability of sexual abstinence should be evaluated in relation to the duration of the clinical trial and the preferred and usual lifestyle of the patient. Periodic abstinence (e.g., calendar, ovulation, symptothermal, or postovulation methods) and withdrawal are not acceptable methods of contraception.
12. 12. For men: agreement to remain abstinent (refrain from heterosexual intercourse) or use contraceptive measures, and agreement to refrain from donating sperm, as defined below:
13. With female partners of childbearing potential, men must remain abstinent or use a condom plus an additional contraceptive method that together result in a failure rate of < 1% per year during the treatment period and for 6 months after the last dose of study treatment. Men must refrain from donating sperm during this same period. With pregnant female partners, men must remain abstinent or use a condom

#### Exclusion Criteria

1. Prior systemic therapy of metastatic disease. Note: Prior adjuvant chemotherapy is permitted, if completed > 3 months prior to randomization. Multimodal treatment of rectal cancer is not considered anti- metastatic therapy and does not preclude study participation
2. Known brain metastasis. In case of symptoms that are suggestive of brain metastasis, brain metastasis has to be ruled out by means of cranialCT/MRI.
3. Significant cardiovascular disease such as: New York Heart Association Class III or greater heart failure; myocardial infarction within 6 months prior to randomization; balloon angioplasty (PTCA) with or without stenting within 6 months prior to randomization; despite anti-arrhythmic therapy unstable cardiac arrhythmia > grade 2 NCI-CTCAE; unstable angina pectoris
4. Transient ischaemic attack or cerebrovascular accident within 6 months prior to randomization, history of cerebral or aortic aneurysm or dissection
5. Medical history of deep vein thrombosis or pulmonary embolism within 6 months prior to randomization or medical history of recurrent thromboembolic events (> 1 episode of deep vein thrombosis, pulmonary embolism, peripheral embolism) within the last 2 years.
6. Severe bleeding event within the last 6 months before randomization(except tumor bleeding surgically treated by tumor resection)
7. Evidence of bleeding diathesis or significant coagulopathy
8. Uncontrolled hypertension defined as systolic blood pressure ≥ 160 mmHg and/or diastolic ≥100 mmHg under antihypertensive medication
9. Severe chronic non-healing wounds, ulcerous lesions or untreated bone fracture.
10. History of abdominal or tracheoesophageal fistula or gastrointestinalperforation, or intra-abdominal abscess -unrelated to surgery- within 6 months prior to randomization.
11. Acute or subacute bowel obstruction, active chronic inflammatory boweldisease or chronic diarrhea
12. History of keratitis, ulcerative keratitis or severe dry eye.
13. Hypersensitivity to trifluridine/tipiracil or panitumumab or bevacizumab or anyof the excipients, known hypersensitivity to Chinese hamster ovary cell products, known hypersensitivity to human or humanized antibodies
14. Current or recent (within 10 days of randomization) use of or anticipated need for continuous treatment during study treatment with acetylsalicylic acid > 325 mg/day or treatment with dipyramidole, ticlopidine > 2 × 250 mg/day, clopidogrel > 75 mg/day, and cilostazol. Combination of these drugs are not allowed.
15. Major surgical procedure, open biopsy, or significant traumatic injury within 28 days prior to randomization, or abdominal surgery, abdominal interventions or significant abdominal traumatic injury within 28 days prior to randomization or anticipation of need for major surgical procedure during the course of the study or non-recovery from side effects of any such procedure
16. Core biopsy or other minor surgical procedure, excluding placement of a vascular access devices, within 3 days prior to the first dose of bevacizumab
17. History of idiopathic pulmonary fibrosis, organizing pneumonia (e.g.,bronchiolitis obliterans), drug-induced pneumonitis/interstitial pneumonia, or idiopathic pneumonitis/interstitial pneumonia, or evidence of active pneumonitis or pulmonary fibrosis on screening chest imaging 1
18. Any other disease, metabolic dysfunction, physical examination finding, or clinical laboratory finding that contraindicates the use of an investigational drug, may affect the interpretation of the results, or may render the patient at high risk from treatment complications.
19. Medical history of other malignant disease than mCRC with the following exceptions: - patients who have been disease-free for at least three years before randomization - patients with adequately treated and completely resected basal cell or squamous cell skin cancer, in situ cervical, breast or prostate cancer, stageI uterine cancer – patients with any treated or untreated malignant disease that is associated with a 5 year survival prognosis of ≥90% and does not require active therapy
20. Known alcohol or drug abuse
21. Pregnant or breastfeeding females
22. Participation in a clinical trial or experimental drug treatment within 28 days prior to inclusion in the clinical trial or within a period of 5 half-lives of the substances administered in a clinical trial or during an experimental drug treatment prior to inclusion in the clinical trial, depending on which period is longest, or simultaneous participation in another clinical trial while taking part in this clinical trial.
23. Patient committed to an institution by virtue of an order issued either bythe judicial or the administrative authorities
24. Patient possibly dependent from the investigator including the spouse, children and close relatives of any investigator
25. Limited legal capacity

### Treatment, dosage and administration

Eligible patients with mCRC will receive first-line treatment with trifluridine/tipiracil plus either panitumumab or bevacizumab in 28-day cycles (Table 2).

**Table 2:** Investigational medicinal products used in the FIRE-8 trial during first-line treatment

Study Arm	IMP	Dose	Dosing schedule (day of 28-day cycle)	Administration
A and B	Trifluridine/ tipiracil	35 mg/m^2^/dose BID	1–5 and 8–12	orally
A	Panitumumab	6 mg/kg body weight	1 and 15	i.v. over 30–60 min^a^
B	Bevacizumab	5 mg/kg body weight	1 and 15	i.v. over 60 ± 15 min^b^

*IMP* Investigational medicinal product, *i.v.* intravenous infusion, *BID* twice daily

^a^first administration over 60 min; ^b^first administration over 90 ± 15 min

#### Arm A

Trifluridine/tipiracil is administered at a dose of 35 mg/m^2^/dose orally, twice daily for five days with two days rest for two weeks (days 1–5 and 8–12) followed by a 14-day rest, repeated every 4 weeks (28-day cycle). Panitumumab is administered at a dose of 6 mg/kg body weight as an intravenous infusion over the course of 30 to 60 min on day 1 and 15 (first administration over 60 min).

#### Arm B

Trifluridine/tipiracil is administered at a dose of 35 mg/m^2^/dose orally, twice daily for five days with two days rest for two weeks (days 1–5 and 8–12) followed by a 14-day rest, repeated every 4 weeks (28-day cycle). Bevacizumab is administered at a dose of 5 mg/kg body weight as an intravenous infusion over the course of 60 ± 15 min on day 1 and 15 (first administration over 90 ± 15 min).

In both treatment arms the dosage of trifluridine/tipiracil is calculated according to body surface area (BSA) as displayed in Table 3. The dosage must not exceed 80 mg/dose. If any doses of trifluridine/tipiracil are missed, the patient is not allowed to make up for missed doses.

**Table 3:** Dose calculation of trifluridine/tipiracil according to BSA

Dose (mg/m^2^)	BSA (m^2^)	Dose in mg (BID)	Tablets per Dose (BID) 15 mg/6.14 mg	Tablets per Dose (BID) 20 mg/8.19 mg	Total daily dose (mg)
35	< 1.07	35	1	1	70
	1.07–1.22	40	0	2	80
	1.23–1.37	45	3	0	90
	1.38–1.52	50	2	1	100
	1.53–1.68	55	1	2	110
	1.69–1.83	60	0	3	120
	1.84–1.98	65	3	1	130
	1.99–2.14	70	2	2	140
	2.15–2.29	75	1	3	150
	≥ 2.30	80	0	4	160

*BSA* Body surface area. *BID* twice daily

#### Dose modifications and dose delays

Doses for trifluridine/tipiracil as well as for panitumumab will be reduced or discontinued temporarily or permanently for specified haematological and non-haematological adverse events. Dose modifications for bevacizumab are not allowed. In case of bevacizumab-related adverse event, the administration can be interrupted or permanently discontinued. In case of concurrent toxicities, dose adjustments are to be made according to the toxicity requiring the furthest dose level reduction.

Administration of trifluridine/tipiracil in any new treatment cycle may only be started if the following requirements are fulfilled:

- Absolute neutrophil count ≥1.5 × 10^9^/L
- Platelets ≥75 × 10^9^/L
- Any CTCAE v.5 grade ≥ 3 non-haematological toxicity causally related to trifluridine/ tipiracil has resolved to grade 1 or baseline grade.

In case of hematological or non-hematological toxicities associated with trifluridine/tipiracil, the criteria for dose interruption and dose modification according to Table 4 will apply.

**Table 4:** Guidelines for management of adverse reactions associated with trifluridine/tipiracil

Adverse reaction	Measure
• Absolute neutrophil count < 0.5 × 10^9^/L	• Interrupt dosing until absolute neutrophil count ≥1.5 × 10^9^/L
• Platelet count < 50 × 109/L	• Interrupt dosing until platelet count ≥75 × 10^9^/L
• Febrile neutropenia	• Interrupt dosing until toxicity resolves to grade 1 or baseline. • When resuming dosing, decrease the dose level by 5 mg/m^2^/dose from the previous dose level. • Do not increase dose after it has been reduced.
• CTCAE v.5 grade 4 neutropenia (< 0.5 × 10^9^/L) or thrombocytopenia (< 25 × 10^9^/L) that results in more than one week’s delay in start of next cycle	
• CTCAE v.5 non-haematologic grade 3 or grade 4 adverse reaction; except for grade nausea and/or vomiting controlled by antiemetic therapy or diarrhoea responsive to antidiarrhoeal medicinal products	

If the planned treatment with trifluridine/tipiracil is delayed by more than six weeks before the start of a new cycle or interrupted by more than six weeks during a treatment cycle, trifluridine/tipiracil has to be discontinued permanently. If the planned treatment with panitumumab or bevacizumab is delayed by more than six weeks (counted from the planned treatment date on Day 1 or Day 15 of a cycle), panitumumab or bevacizumab has to be discontinued permanently.

In case trifluridine/tipiracil has to be permanently discontinued due to unacceptable toxicity within treatment arm A, panitumumab may be continued as a single agent (investigator’s decision) until progression according to RECIST 1.1 or occurrence of unacceptable toxicity. If panitumumab has to be permanently discontinued due to unacceptable toxicity, treatment with trifluridine/tipiracil might be continued as monotherapy (investigator’s decision) until progression according to RECIST 1.1 or occurrence of unacceptable toxicity to trifluridine/tipiracil.

In case trifluridine/tipiracil has to be permanently discontinued due to unacceptable toxicity within treatment arm B, study treatment needs to be discontinued. The administration of bevacizumab as a monotherapy is not allowed according to protocol. If bevacizumab has to be permanently discontinued due to unacceptable toxicity, treatment with trifluridine/tipiracil might be continued as monotherapy (investigator’s decision) until disease progression according to RECIST 1.1 or occurrence of unacceptable toxicity due to trifluridine/tipiracil.

##### Dose modification of trifluridine/tipiracil

A maximum of three dose reduction levels (30 mg/m^2^ BSA BID, 25 mg/m^2^ BSA BID, and 20 mg/m^2^ BSA BID) are permitted for trifluridine/tipiracil to a minimum dose of 20 mg/m^2^ BID trifluridine/tipiracil. Table 5 displays the dose in mg to be taken twice daily calculated per BSA and the necessary number of 15 mg/6.14 mg and 20 mg/ 8.19 mg tablets trifluridine/tipiracil to be taken.

**Table 5:** Dose calculation of trifluridine/tipiracil according to BSA for dose reduction levels

Dose (mg/m^2^)	BSA (m^2^)	Dose in mg (BID)	Tablets per Dose (BID) 15 mg/6.14 mg	Tablets per Dose (BID) 20 mg/8.19 mg	Total daily dose (mg)
**Level 1 dose reduction: From 35 mg/m**^**2**^ **to 30 mg/m**^**2**^					
30	< 1.09	30	2	0	60
	1.09–1.24	35	1	1	70
	1.25–1.39	40	0	2	80
	1.40–1.54	45	3	0	90
	1.55–1.69	50	2	1	100
	1.70–1.94	55	1	2	110
	1.95–1.09	60	0	3	120
	2.10–2.28	65	3	1	130
	≥ 2.29	70	2	2	140
**Level 2 dose reduction: From 30 mg/m**^**2**^ **to 25 mg/m**^**2**^					
25	< 1.10	25 ^a^	2 ^a^	1 ^a^	50
	1.10–1.29	30	2	0	60
	1.30–1.49	35	1	1	70
	1.50–1.69	40	0	2	80
	1.70–1.89	45	3	0	90
	1.90–2.09	50	2	1	100
	2.10–2.29	55	1	2	110
	≥ 2.30	60	0	3	120
**Level 3 dose reduction: From 25 mg/m**^**2**^ **to 20 mg/m**^**2**^					
20	< 1.14	20	0	1	40
	1.14–1.34	25^a^	2 ^a^	1 ^a^	50
	1.35–1.59	30	2	0	60
	1.60–1.94	35	1	1	70
	1.95–2.09	40	0	2	80
	2.10–2.34	45	3	0	90
	≥ 2.35	50	2	1	100

*BSA* Body surface area, *BID* twice daily

^a^At a total daily dose of 50 mg, patients should take 1 × 20 mg /8.19 tablet in the morning and 2 × 15 mg/6.14 mg tablets in the evening

##### Dose modification of panitumumab

The majority of anti-EGFR-related dermatologic adverse reactions are mild to moderate. If a patient develops CTCAE grade ≥ 3 dermatologic reactions, soft tissue toxicity or reactions that are considered intolerable, the following dose modification are recommended (Table 6).

**Table 6:** Dose modifications for panitumumab in case of CTCAE grade ≥ 3 skin or soft tissue toxicity

Occurrence of skin symptom(s): CTCAE grade ≥ 3	Administration of panitumumab	Outcome	Dose regulation
Initial occurrence	Withhold 1 or 2 doses	Improved (CTCAE grade < 3)	Continue at 100% of original dose (6 mg/kg)
		Not recovered	Discontinue panitumumab
At the second occurence	Withhold 1 or 2 doses	Improved (CTCAE grade < 3)	Continue at 80% of original dose (4.8 mg/kg)
		Not recovered	Discontinue panitumumab
At the third occurence	Withhold 1 or 2 doses	Improved (CTCAE grade < 3)	Continue at 60% of original dose (3.6 mg/kg)
		Not recovered	Discontinue panitumumab
At the fourth occurence	Discontinue panitumumab		

#### Concomitant medication

Any treatments and medication that are considered necessary for the patient’s welfare according to the investigator and will not interfere with the clinical trial may be given. All concomitant medication must be recorded in the patients source data and the eCRF.

Use of the following concomitant therapies is prohibited: Any therapy intended for the systemic treatment of cancer (e.g. chemotherapy, hormonal therapy, immunotherapy, and herbal therapy) other than study treatment. Investigational therapy is prohibited within 28 days prior to study randomization.

##### Statistical considerations

The study is a prospective, randomized, open-label, multicenter phase II study with two parallel arms to investigate the efficacy, patient reported QoL and safety of trifluridine/tipiracil in combination with panitumumab (arm A) versus trifluridine/tipiracil plus bevacizumab (arm B) as first-line treatment of mCRC.

#### Statistical Hypothesis and Sample Size Determination

The primary endpoint will be tested to demonstrate superiority of initial treatment with trifluridine/tipiracil plus panitumumab (arm A) versus trifluridine/tipiracil plus bevacizumab (arm B). Patients are randomized in a 1:1 ratio into arm A and arm B.

For arm B, an objective response rate of 30% is assumed based on previous studies [4, 6, 7]. For arm A, we hypothesize an improvement in the objective response rate of 25% leading to an estimated response rate of 55% based on the results of the PANDA trial [8]. This difference corresponds to an odds ratio of 2.85.

A Fisher’s exact test with a two-sided nominal significance level of 0.05 will have at least 80% power to detect a significant difference when the sample size amounts to 138 patients. Given an estimated drop-out rate of 10% (i.e. patients who received no treatment within the study), 153 patients need to be enrolled.

Secondary and exploratory endpoints will be analyzed in descriptive manner. All additional *p*-values will be estimated exploratorily without adjustment for the level of significance using two-sided test procedures. Demographic and prognostic baseline measures will be analyzed for heterogeneity between the two treatment arms.

#### Analyzed populations

The full analysis set (FAS) represents the main population for the description of baseline characteristics as well as efficacy parameters including the analysis of the primary and secondary endpoints. A sensitivity analysis of the efficacy parameters will be performed in the per-protocol population (PP), which forms a subgroup of the FAS. The PP will only comprise of the FAS patients, excluding patients who meet at least one of the following criteria:

- Administration of only one cycle of treatment with study medication trifluridine/tipiracil plus bevacizumab or trifluridine/tipiracil plus panitumumab
- Any severe violation of inclusion or exclusion criteria (decided as blinded-to-the-arm assessment by the sponsor)
- Severe protocol violation (if necessary a data review meeting will decide which protocol violations require exclusion from the PP set.)

The safety population (SAF) includes patients receiving at least one dose of trifluridine/tipiracil or bevacizumab or panitumumab.

#### Statistical Analysis

The recorded baseline, efficacy, quality of life, and safety data will be presented using standard descriptive methods. For continuous data, distribution parameters (mean, standard deviation, minimum, median, and maximum) will be computed. For categorical data, frequency counts will be given.

The primary endpoint will be tested by means of Fisher’s exact test at a two-sided significance level of 5%. This means that statistical significance is reached with a two-tailed *p* < 0.05. A further sensitivity analysis will be done taken into account the stratification parameters at the time of randomization. Both results will be critically discussed on a comparative basis. The same analysis will be done with the PP set. With regard to response rates (or other rates), patients in whom the respective response criteria are not met will be evaluated as non-responders. Concerning the primary endpoint ORR, patients in the FAS not demonstrating CR or PR will be evaluated as non-responders.

Percentage values (e.g. response and toxicity rates) at specific time points will be calculated with their exact confidence intervals and, if necessary, compared with regard to manifestation and extent by means of appropriate tests (chi-squared test, Fisher’s exact test or Mantel–Haenszel test (or COCHRAN/ ARMITAGE trend test) and with the corresponding stratified variants, respectively. Continuous data will be analyzed using the Wilcoxon test. Comparisons between different acquisition times will be performed using the Wilcoxon test for related samples. Time-to-event data (e.g. progression–free survival, overall survival) will be calculated according to Kaplan-Meier. Estimates for the median time to event as well as the proportion of patients not having reached the event after appropriate times will be presented and compared using the log-rank test and Cox regression analysis. The starting point will be the day of randomization. Patients without the respective event will be censored.

Safety data analysis includes exposure to investigational products trifluridine/tipiracil, bevacizumab, and panitumumab as well as type, incidence and severity of adverse events, laboratory parameters and laboratory abnormalities. The severity of adverse events will be graded according to CTCAE version 5.0.

The demographic and prognostic baseline data will be tested for homogeneity among treatment groups. In case of major discrepancies in prognostically relevant variables, additional statistical analysis will be performed to adjust for group differences. For multivariate analysis, appropriate regression models e.g. logistic regression model, Cox proportional hazard model will be applied. Missing data will not be replaced. If necessary, incomplete dates will be imputed appropriately.

##### QoL Assessment

QoL analysis will be performed by means of EQ-5D-5L-questionnaires. EQ-5D is a standardized instrument developed by the EuroQol Group as a measure of patient-reported health-related quality of life (HR-QoL).

The EQ-5D consists of a descriptive system and the EQ visual analogue scale (VAS). The descriptive system comprises five dimensions: mobility, self-care, usual activities, pain/discomfort, and anxiety/depression.

The EQ-5D-5L questionnaire will be handed out in paper version at the trial center and completed by the patient prior to any procedures related to the clinical trial. The questionnaires need to be filled out at baseline within 21 days prior to randomization, at the restaging visits about every ten weeks, and at the EOT visit.

##### Assessment of severity/intensity

The investigator is responsible for ensuring that all adverse events are documented in the patients’ source documents and the AE page of the eCRF including the following information: description of AE, date of onset and date of resolution, severity, seriousness, causal relationship to IMP(s), treatments given or actions taken and outcome.

AEs must be recorded from the date of informed consent until 28 days after last administration of any IMP. Following this period, investigators have to report only serious adverse events (SAEs) that are believed to be related to prior treatment with any IMP.

A SAE is defined as any untoward medical occurrence that results in death, is life-threatening, requires inpatient hospitalization or prolongation of existing hospitalization, results in persistent or significant disability/incapacity, a congenital anomaly/birth defect or constitutes an important medical event. Investigators must report all SAEs immediately within 24 hours using the SAE report form.

##### Translational research

The translational research aims to identify and characterize patient subgroups with greatest or lowest benefit from the respective treatment. Among others, correlations of any patient subgroups with response according to radiological imaging criteria (RECIST 1.1) and survival as well as changes in circulating tumor DNA (ctDNA) or inflammation will be investigated.

Copies of CT- and /or MRI images sent for prespecified central review will be used to analyze correlation of radiological criteria with tumor response, course of the tumor disease (for example OS and PFS), type of tumor disease, interaction of patient/tumor characteristics and in further exploratory manners (for example RADIOMICS). All translational research subprojects are studied exclusively in the context of exploratory analysis.

**Fig. 2:**
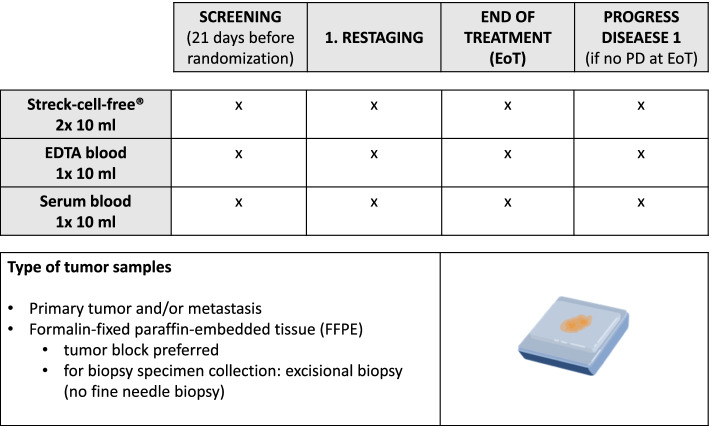
FIRE-8 translational research. Microsoft PowerPoint version 16.62 was used to generate this figure

Residual formalin-fixed, paraffin-embedded (FFPE) tumor tissue samples (primary tumor or metastasis) will be collected to analyze mutations relevant for treatment (e.g. BRAF), gene expression subgroups, and consensus molecular subtypes (CMS) of colorectal cancer. These might also include analysis of germline mutations. Blood samples will be taken to analyze tumor evolution (by means of ctDNA), inflammation- and immunomarkers (Fig. 2).

##### Regulatory, ethical, legal and trial oversight consideration

The study protocol was approved by local ethics committee of the Charité University Medicine Berlin (State Office for Health and Social Affairs – LAGeSo, 21-634_10-Haupt – IV E 10) and the local ethic committees of the participating centers throughout Germany. The clinical trial will be conducted in accordance with the ethical principles of the Declaration of Helsinki and International Council for Harmonisation of Technical Requirements for Pharmaceuticals for Human Use (ICH) Good Clinical Practice guidelines, and the applicable European and domestic law concerning the conduct of clinical studies.

#### Informed consent

Informed consent is the free and voluntary agreement of a patient to participate in a clinical trial after having been informed about all aspects of the clinical trial relevant to the patient’s decision to participate. The investigators must obtain freely given informed consent from every patient prior to any procedure related to the clinical trial.

#### Confidentiality and data protection

The sponsor affirms the patient’s right to protection against invasion of privacy. All pertinent provisions of European and national data protection legislation in order to guarantee confidentiality and protection of privacy will be fully observed.

All records identifying the patients will be kept confidential and, to the extent permitted by theapplicable laws and/or regulations, will not be made publicly available.

All data transfer with the trial centres will be made without any exception via the patient-code. All participating trial centres are obliged to keep a strictly confidential patient identification list at a safe locked place.

#### Monitoring and audits

An Independent Data Monitoring Committee (IDMC) constituting of three oncology experts who are not participating in the study will be monitoring and assessing safety data from this clinical trial. Details will be laid down in an IDMC charter. The IDMC will give recommendations how to proceed with the trial. The results of the interim safety analysis will also be presented to the IDMC. Recruitment of further patients will not be halted until the data of this interim safety analysis are available and the IDMC has given recommendations how to proceed with the trial.

The sponsor may conduct or commission audits in the course of the trial, which are independent of and separate from routine monitoring or quality control functions, to evaluate trial conduct and compliance with the protocol, SOPs, GCP and the applicable regulatory requirements.

## Discussion

The clinical benefit of first-line treatment with combination chemotherapy including oxaliplatin or irinotecan compared to monochemotherapy with FP in elderly patients with mCRC remains controversial. Two phase III clinical trials conducted in elderly mCRC patients compared FP to combination therapy with oxaliplatin (FOCUS 2 trial [15]) and irinotecan (FFCD 2001–02 [16]), respectively, neither suggesting significant improvement in OS or PFS using combination chemotherapy. A subsequent meta-analysis that pooled data from elderly mCRC subgroups across trials found that combination therapy significantly improved PFS (HR 0.82, *P* = 0.003), but not OS (HR 1.00, *P* = 0.962) [17]. A recent japanese trial investigating initial oxaliplatin added to fluoropyrimidine plus bevacizumab (RESPECT) confirmed this perspective: apart from response rates, no benefit in efficacy endpoints was evident [18]. Given the limited evidence of additional benefit from combination chemotherapy along with poorer tolerability, monochemotherapy in combination with monocloncal antibodies appears to be a very reasonable option in elderly mCRC patients as well as in patients that are unwilling or unfit to undergo combination chemotherapy. The administration of the oral FP trifluridine/tipiracil instead of intravenous chemotherapy offers several advantages, especially in elderly patients, including the avoidance of infusion-related complications and an increase in quality of life and autonomy. In addition, trifluridine/tipiracil might be better tolerated compared to other oral fluoropyrimides such as capecitabine, which has a more pronounced side effect profile (e.g. hand-foot syndrome), especially in combination with an anti-EGFR antibody.

Recommendations for the use of monochemotherapy in first-line treatment setting mainly refer to FP based regimens [4, 5, 7, 19]. Following the findings of the phase III RECOURSE trial, which demonstrated promising efficacy, with a favorable safety profile, trifluridine/tipiracil was established for third-line treatment in mCRC patients. A randomized phase II trial suggested that this “further-line” treatment strategy of trifluridine/tipiracil could even be improved by the addition of bevacizumab [20]. Application of trifluridine/tipiracil in first-line treatment is not approved and may not be approved in the future, although clinical trials suggested the efficacy and tolerability in elderly untreated mCRC patients [9, 14, 21]. Multiple studies have demonstrated superiority of adding anti-VEGF antibodies to FP monochemotherapy without occurrence of major toxicity or QoL impairment in previously untreated mCRC patients [4, 5, 7, 19].

The FIRE-8 trial has several limitations. Due to the phase II study design and the correspondingly small sample size (planned enrollment of 153 patients) the data obtained within this trial cannot be considered confirmatory. Therefore, secondary endpoints including OS and PFS will be subjected to exploratory analyses with limited power only. If the new treatment concept proves to be effective, subsequent phase III studies are needed before implementation as a standard of care will be accepted.

The primary endpoint investigated in this trial is overall response rate (ORR) according to RECIST 1.1. Response-based outcomes including ORR represent important surrogate endpoints in phase II trials, determining evidence of anti-tumor activity. In advanced colorectal cancer increase in tumor response rate is known to translate into an increase in overall survival [22], especially in patients with *RAS* wild type [23]. However, classic phase III trial designs usually aim to time-to-event endpoints and therefore the acceptance of improved ORR might be uncertain.

Within this trial, primary tumor sidedness, as well as BRAF V600E mutation status, are not considered among inclusion/exclusion criteria, since the corresponding labelling of panitumumab has no restriction concerning BRAF mutation and/or sidedness of the primary tumor. Current retrospective data implicate evidence of a lack of benefit from anti-EGFR treatment in right-sided tumors regarding PFS and OS [12, 13], while limited, if any, benefit was reported in terms of ORR [13]- therefore not interfering with the primary endpoint of this trial. Further, prospective validation of these results is still pending and chemotherapy plus anti-EGFR antibody remains a labelled treatment option in this patient population, although randomization into FIRE-8 might be a suboptimal choice for the majority of patients with right-sided primary tumor and/or *BRAF* V600E mutant mCRC [12, 13, 24]. By contrast, it might be argued that defined circumstances may support trifluridine/tipiracil in these respective patients (i.e. inability to receive doublet chemotherapy and anti-VEGF agents, missing data to which extent interaction of tumor location and *BRAF* mutation may interact with anti-EGFR therapy in the context of trifluridine/tipiracil). However, during the conduct of the trial, information on primary tumor location and *BRAF* V600E mutation will be collected and considered for subgroup analyses to further clarify this issue.

Based on the results of the phase II TASCO study [9], reporting a trend towards a survival benefit of trifluridine/tipiracil plus bevacizumab compared to standard regimen FP plus bevacizumab in untreated mCRC patients, we decided to further investigate the efficacy of trifluridine/tipiracil plus bevacicumab within the FIRE-8 trial. Even though recently published data of the subsequent phase III study SOLSTICE failed to demonstrate postulated superiority of the experimental treatment concept, a favourable trend toward better efficacy in the trifluridine/tipiracil plus bevacizumab arm was evident, and further evaluation of this combination and other regimens with trifluridine/tipiracil plus anti-EGFR (i.e. panitumumab) appears warranted.

## Conclusion

In metastatic colorectal cancer, treatment options for patients ineligible or unwilling to undergo intensive chemotherapy regimens are still limited. Taking into consideration that more than half of patients diagnosed with CRC are 70 years of age and older accompanying comorbidities, there is a need for treatment options beyond the current standard of care. To our knowledge, the FIRE-8 trial is the first study to evaluate the efficacy of trifluridine/tipiracil plus panitumumab in comparison with trifluridine/tipiracil plus bevacizumab in untreated *RAS* WT mCRC patients. The trial is designed to provide an additional reasonable treatment option integrating an anti-EGFR antibody for untreated *RAS* WT mCRC patients.

## Supplementary Information

**Additional file 1.** Schedule of Study Assessments for the FIRE-8 trial.

## Data Availability

Not applicable.

## Abbreviations

AE: Adverse event
BID: Twice daily
BSA: Body Surface Area
BW: Body Weight
CRC: Colorectal cancer
ctDNA: Circulating Tumor DNA
DpR: Depth of response
ECOG: Eastern Cooperative Oncology Group
EGFR: Epidermal Growth Factor Receptor
EoT: End of Treatment
ETS: Early tumor shrinkage
FAS: Full Analysis Set
FFPE: Formalin-Fixed Paraffin-Embedded
FP: Fluoropryrimine
GCP: Good Clinical Practice
IDMC: Independent Data Monitoring Committee
IMP: Investigational medicinal product
INR: International Normalized Ratio
IRI: Irinotecan
mCRC: Metastatic Colorectal Cancer
mCRC: Metastatic colorectal cancer
ORR: Objective response rate
OS: Overall survival
OX: Oxaliplatin
PFS: Progression-free survival
PP: Per-Protocol Population
PTT: Partial Thromboplastin Time
QoL: Quality of Life
RECIST: Response evaluation criteria in solid tumors
SAE: Severe adverse event
SAF: Safety population
SOP: Standard Operating Procedure
ULN: Upper Limit of Normal
WT: Wild-type

## References

[1] Siegel RL, Miller KD, Goding Sauer A, Fedewa SA, Butterly LF, Anderson JC, Cercek A, Smith RA, Jemal A (2020). Colorectal cancer statistics, 2020. CA Cancer J Clin.

[2] Benson AB, Venook AP, Cederquist L, Chan E, Chen YJ, Cooper HS, Deming D, Engstrom PF, Enzinger PC, Fichera A (2017). Colon Cancer, Version 1.2017, NCCN Clinical Practice Guidelines in Oncology. J Natl Compr Cancer Netw.

[3] Van Cutsem E, Cervantes A, Adam R, Sobrero A, Van Krieken JH, Aderka D, Aranda Aguilar E, Bardelli A, Benson A, Bodoky G (2016). ESMO consensus guidelines for the management of patients with metastatic colorectal cancer. Ann Oncol.

[4] Cunningham D, Lang I, Marcuello E, Lorusso V, Ocvirk J, Shin DB, Jonker D, Osborne S, Andre N, Waterkamp D (2013). Bevacizumab plus capecitabine versus capecitabine alone in elderly patients with previously untreated metastatic colorectal cancer (AVEX): an open-label, randomised phase 3 trial. Lancet Oncol.

[5] Kabbinavar FF, Hambleton J, Mass RD, Hurwitz HI, Bergsland E, Sarkar S (2005). Combined analysis of efficacy: the addition of bevacizumab to fluorouracil/leucovorin improves survival for patients with metastatic colorectal cancer. J Clin Oncol.

[6] Kabbinavar FF, Hurwitz HI, Yi J, Sarkar S, Rosen O (2009). Addition of bevacizumab to fluorouracil-based first-line treatment of metastatic colorectal cancer: pooled analysis of cohorts of older patients from two randomized clinical trials. J Clin Oncol.

[7] Tebbutt NC, Wilson K, Gebski VJ, Cummins MM, Zannino D, van Hazel GA, Robinson B, Broad A, Ganju V, Ackland SP (2010). Capecitabine, bevacizumab, and mitomycin in first-line treatment of metastatic colorectal cancer: results of the Australasian Gastrointestinal Trials Group Randomized Phase III MAX Study. J Clin Oncol.

[8] Lonardi S, Schirripa M, Buggin F, Antonuzzo L, Merelli B, Boscolo G, Cinieri S, Di Donato S, Lobefaro R, Moretto R (2020). First-line FOLFOX plus panitumumab versus 5FU plus panitumumab in RAS-BRAF wild-type metastatic colorectal cancer elderly patients: The PANDA study. J Clin Oncol.

[9] Van Cutsem E, Danielewicz I, Saunders MP, Pfeiffer P, Argilés G, Borg C, Glynne-Jones R, Punt CJA, Van de Wouw AJ, Fedyanin M (2020). Trifluridine/tipiracil plus bevacizumab in patients with untreated metastatic colorectal cancer ineligible for intensive therapy: the randomized TASCO1 study. Ann Oncol.

[10] Mayer RJ, Van Cutsem E, Falcone A, Yoshino T, Garcia-Carbonero R, Mizunuma N, Yamazaki K, Shimada Y, Tabernero J, Komatsu Y (2015). Randomized trial of TAS-102 for refractory metastatic colorectal cancer. N Engl J Med.

[11] André T, Falcone A, Shparyk Y, Moiseenko FV, Marques E, Csoszi T, et al. VP11-2021: Trifluridine/tipiracil plus bevacizumab vs capecitabine plus bevacizumab as first line treatment for patients with metastatic colorectal cancer (mCRC) ineligible for intensive therapy: The phase III randomized SOLSTICE study. Ann Oncol. 2022;33(2): 229–30.

[12] Holch JW, Ricard I, Stintzing S, Modest DP, Heinemann V (2017). The relevance of primary tumour location in patients with metastatic colorectal cancer: a meta-analysis of first-line clinical trials. Eur J Cancer.

[13] Arnold D, Lueza B, Douillard JY, Peeters M, Lenz HJ, Venook A, Heinemann V, Van Cutsem E, Pignon JP, Tabernero J (2017). Prognostic and predictive value of primary tumour side in patients with RAS wild-type metastatic colorectal cancer treated with chemotherapy and EGFR directed antibodies in six randomized trials. Ann Oncol.

[14] Kuboki Y, Yoshino T, Kato T, Kagawa Y, Gamoh M, Yasui H, Yamazaki K, Komatsu Y, Satake H, Goto M (2018). APOLLON: A phase I/II study of panitumumab combined with TAS-102 in patients (pts) with RAS wild-type (wt) metastatic colorectal cancer (mCRC). J Clin Oncol.

[15] Seymour MT, Thompson LC, Wasan HS, Middleton G, Brewster AE, Shepherd SF, O'Mahony MS, Maughan TS, Parmar M, Langley RE (2011). Chemotherapy options in elderly and frail patients with metastatic colorectal cancer (MRC FOCUS2): an open-label, randomised factorial trial. Lancet.

[16] Aparicio T, Lavau-Denes S, Phelip JM, Maillard E, Jouve JL, Gargot D, Gasmi M, Locher C, Adhoute X, Michel P (2016). Randomized phase III trial in elderly patients comparing LV5FU2 with or without irinotecan for first-line treatment of metastatic colorectal cancer (FFCD 2001-02). Ann Oncol.

[17] Landre T, Uzzan B, Nicolas P, Aparicio T, Zelek L, Mary F, Taleb C, Des Guetz G (2015). Doublet chemotherapy vs. single-agent therapy with 5FU in elderly patients with metastatic colorectal cancer. a meta-analysis. Int J Color Dis.

[18] Hamaguchi T, Takashima A, Mizusawa J, Shimada Y, Nagashima F, Ando M, Ojima H, Denda T, Watanabe J, Shinozaki K (2022). A randomized phase III trial of mFOLFOX7 or CapeOX plus bevacizumab versus 5-FU/l-LV or capecitabine plus bevacizumab as initial therapy in elderly patients with metastatic colorectal cancer: JCOG1018 study (RESPECT). J Clin Oncol.

[19] Botrel TEA, Clark LGO, Paladini L, Clark OAC (2016). Efficacy and safety of bevacizumab plus chemotherapy compared to chemotherapy alone in previously untreated advanced or metastatic colorectal cancer: a systematic review and meta-analysis. BMC Cancer.

[20] Pfeiffer P, Yilmaz M, Möller S, Zitnjak D, Krogh M, Petersen LN, Poulsen L, Winther SB, Thomsen KG, Qvortrup C (2020). TAS-102 with or without bevacizumab in patients with chemorefractory metastatic colorectal cancer: an investigator-initiated, open-label, randomised, phase 2 trial. Lancet Oncol.

[21] Oki E, Makiyama A, Miyamoto Y, Kotaka M, Kawanaka H, Miwa K, Kabashima A, Noguchi T, Yuge K, Kashiwada T (2021). Trifluridine/tipiracil plus bevacizumab as a first-line treatment for elderly patients with metastatic colorectal cancer (KSCC1602): A multicenter phase II trial. Cancer Med.

[22] Buyse M, Thirion P, Carlson RW, Burzykowski T, Molenberghs G, Piedbois P (2000). Relation between tumour response to first-line chemotherapy and survival in advanced colorectal cancer: a meta-analysis**.** Meta-Analysis Group in Cancer. Lancet.

[23] De Roock W, Piessevaux H, De Schutter J, Janssens M, De Hertogh G, Personeni N, Biesmans B, Van Laethem JL, Peeters M, Humblet Y (2008). KRAS wild-type state predicts survival and is associated to early radiological response in metastatic colorectal cancer treated with cetuximab. Ann Oncol.

[24] Stintzing S, Heinrich K, Tougeron D, Modest DP, Schwaner I, Euker J, Pihusch R, Stauch M, Kaiser F, Kahl C (2021). Randomized study to investigate FOLFOXIRI plus either bevacizumab or cetuximab as first-line treatment of BRAF V600E-mutant mCRC: The phase-II FIRE-4.5 study (AIO KRK-0116). J Clin Oncol.

