# Supplementary material for: Study protocol of the FIRE-8 (AIO-KRK/YMO-0519) trial: a prospective, randomized, open-label, multicenter phase II trial investigating the efficacy of trifluridine/tipiracil plus panitumumab versus trifluridine/tipiracil plus bevacizumab as first-line treatment in patients with metastatic colorectal cancer
Source: BMC Cancer. 2022 Jul 27;22:820. doi: 10.1186/s12885-022-09892-8 (PMC9327141; doi:10.1186/s12885-022-09892-8)
Supplement: Supplementary file 1 — Additional file 1. Schedule of Study Assessments for the FIRE-8 trial. [file 12885_2022_9892_MOESM1_ESM.docx]

### Additional file 1: Schedule of Study Assessments for the FIRE-8 trial

|  | **Screening Procedures/ Baseline Assessments** | **Treatment phase** | | | **End of study treatment (EoT) visit** | **Follow-up** |
| --- | --- | --- | --- | --- | --- | --- |
| Assessment Window | Within 35 days before randomisation (unless otherwise indicated), assessments from the clinical routine (pre-screening phase) can be used for the trial if they meet the timelines | On Day 12 of each cycle before administration of study medication or within three days before | On Day 152 of each cycle before administration of study medication or within three days before | Restaging:  Every 10 weeks ± 14 days  and upon progression on study treatment | At 28 ± 14 days after the last administration of IMP | Every 3 months ± 2 weeks after the end of treatment (EoT) visit. Follow up until 5 years after randomisation or death |
| Declaration of consent1 | x |  |  |  |  |  |
| Verification of inclusion and exclusion criteria | x |  |  |  |  |  |
| Medical history mCRC6 | x |  |  |  |  |  |
| Medical history of prior and concomitant diseases/ disorders, demography including age and self- reported ethnicity | x |  |  |  |  |  |
| Complete physical examination, height, body weight7 | x |  |  |  |  |  |
| Limited physical examination8, body weight |  | x | x |  | x |  |

|  | **Screening Procedures/ Baseline Assessments** | **Treatment phase** | | | **End of study treatment (EoT) visit** | **Follow-up** |
| --- | --- | --- | --- | --- | --- | --- |
| Assessment Window | Within 35 days before randomisation (unless otherwise indicated), assessments from the clinical routine (pre-screening phase) can be used for the trial if they meet the timelines | On Day 12 of each cycle before administration of study medication or within three days before | On Day 152 of each cycle before administration of study medication or within three days before | Restaging:  Every 10 weeks ± 14 days  and upon progression on study treatment | At 28 ± 14 days after the last administration of IMP | Every 3 months ± 2 weeks after the end of treatment (EoT) visit. Follow up until 5 years after randomisation or death |
| Vital signs (blood pressure, pulse) | x4 | x | x | x | x |  |
| ECOG performance status | x | x15 |  |  | x |  |
| 12-lead ECG | x |  |  |  | x |  |
| Tumor assessment according to RECIST version 1.1 | x |  |  | x | (x)^11^ | (x) |
| Abdominal CT/MRI9 | x |  |  | x | (x)^11^ | (x)^12^ |
| Chest CT | x |  |  | (x)^10^ | (x)10, 11 | (x)10, 12 |
| Cranial CT or MRI (only in case of suspected CNS metastases) | (x) |  |  | (x) | (x)^11^ | (x)^12^ |

|  | **Screening Procedures/ Baseline Assessments** | **Treatment phase** | | | **End of study treatment (EoT) visit** | **Follow-up** |
| --- | --- | --- | --- | --- | --- | --- |
| Assessment Window | Within 35 days before randomisation (unless otherwise indicated), assessments from the clinical routine (pre-screening phase) can be used for the trial if they meet the timelines | On Day 12 of each cycle before administration of study medication or within three days before | On Day 152 of each cycle before administration of study medication or within three days before | Restaging:  Every 10 weeks ± 14 days  and upon progression on study treatment | At 28 ± 14 days after the last administration of IMP | Every 3 months ± 2 weeks after the end of treatment (EoT) visit. Follow up until 5 years after randomisation or death |
| Other imaging examinations if appropriate in case of suspected progression/metastasis | (x) |  |  | (x) | (x)^11^ | (x)^12^ |
| Documentation of *RAS* wild-type, determined by means of validated test method (local pathology) | x5 |  |  |  |  |  |
| Haemogram and diff. blood count13, 17 | x4 | x15 | x |  | x |  |
| Clinical chemistry14, 17 | x4 | x15 | x |  | x |  |
| Urinalysis by dipstick16, 17 | x3, 16 | x15 |  |  | x |  |
| CEA 17 | x |  |  | x | (x)^11^ |  |

|  | **Screening Procedures/ Baseline Assessments** | **Treatment phase** | | | **End of study treatment (EoT) visit** | **Follow-up** |
| --- | --- | --- | --- | --- | --- | --- |
| Assessment Window | Within 35 days before randomisation (unless otherwise indicated), assessments from the clinical routine (pre-screening phase) can be used for the trial if they meet the timelines | On Day 12 of each cycle before administration of study medication or within three days before | On Day 152 of each cycle before administration of study medication or within three days before | Restaging:  Every 10 weeks ± 14 days  and upon progression on study treatment | At 28 ± 14 days after the last administration of IMP | Every 3 months ± 2 weeks after the end of treatment (EoT) visit. Follow up until 5 years after randomisation or death |
| Pregnancy test **(only in FCBPs)**; beta HCG in blood or urine permitted17 | x3 | x15 |  |  | x |  |
| QoL questionnaire EQ-5D- 5L | x4,18 |  |  | x | x |  |
| Archival FFPE tumor tissue sample (primary tumor or metastasis permitted) for Translational Research19 | (x) |  |  |  |  |  |
| Blood for Translational Research20 | x4, 20 |  |  | (x)^20^ | (x)^20^ |  |
| Adverse events | Continuously from date of signing informed consent to EoT visit | | | | |  |
| Concomitant medication21 | x21 | Continuously until the EoT visit | | | |  |
| Documentation of survival data |  |  |  |  |  | x |

|  | **Screening Procedures/ Baseline Assessments** | **Treatment phase** | | | **End of study treatment (EoT) visit** | **Follow-up** |
| --- | --- | --- | --- | --- | --- | --- |
| Assessment Window | Within 35 days before randomisation (unless otherwise indicated), assessments from the clinical routine (pre-screening phase) can be used for the trial if they meet the timelines | On Day 12 of each cycle before administration of study medication or within three days before | On Day 152 of each cycle before administration of study medication or within three days before | Restaging:  Every 10 weeks ± 14 days  and upon progression on study treatment | At 28 ± 14 days after the last administration of IMP | Every 3 months ± 2 weeks after the end of treatment (EoT) visit. Follow up until 5 years after randomisation or death |
| Documentation of second- line treatment and subsequent treatment lines22 |  |  |  |  |  | x |

1 Declaration has to be signed prior to any trial-specific examinations or procedures.

2 Scheduled assessments have to be performed before the administration of panitumumab and bevacizumab.

3 Within 14 days before randomisation

4 Within 21 days before randomisation

5 Without predefined time interval before randomisation (i.e. >35 days before randomisation permitted)

6 Date of first diagnosis of CRC and date of diagnosis of metastatic disease; TNM stage and grading at first diagnosis/current TNM stage; primary tumor site and sidedness of the primary tumor; site/sites of metastasis; type of metastases (synchronous metastases or metachronous metastases); histological confirmation; surgery of primary tumor and/or metastases; adjuvant chemotherapy (if any); prior radiotherapy or radiochemotherapy (if any).

7 A complete physical examination required at screening. New or worsened abnormalities should be recorded as adverse events if appropriate.

8 A limited physical examination will be performed at other visits after screening to assess changes from baseline abnormalities and any new abnormalities and to evaluate patient reported symptoms. New or worsened abnormalities should be recorded as adverse events if appropriate.

9 The examination method applied at baseline (CT or MRI) must be continued throughout the entire study

10 Only in case of suspected pulmonary metastasis/known pulmonary metastasis

11 Only required if not done within the last 28 days.

12 Only required if study treatment was discontinued permanently without occurrence of progression, e.g. due to unacceptable toxicity and only required until first occurrence of progression after end of study treatment or start of subsequent anti-tumor treatment-line, whichever date is earlier.

13 Haemogram and differential blood count includes leukocytes, neutrophils, lymphocytes, thrombocytes, and haemoglobin (automated determination)

14 At screening: creatinine and/or calculated creatinine clearance according to Cockcroft and Gault or MDRD, LDH, total bilirubin, ALAT, ASAT, AP, potassium, calcium, magnesium and CRP.

During treatment phase and at EOT visit: Creatinine and/or calculated creatinine clearance according to Cockcroft and Gault or MDRD, ALAT, AP, total bilirubin. Furthermore determination of LDH, ASAT, potassium, calcium, magnesium (Arm A with administration of panitumumab), CRP if clinically indicated.

15 Screening hematology, serum chemistry, pregnancy testing, urinalysis, assessment of ECOG performance status obtained ≤ 5 days prior to the initiation of study treatment do not have to be repeated for Cycle 1 Day 1.

16 Urinalysis by urine dipstick including specific gravity, pH, glucose, protein, ketones, and blood. (**Note**: If the urine dipstick indicates ≥ ++ for proteinuria at

screening, an additional 24-hour urine collection is required to demonstrate < 1 g of protein in 24 hours to meet the inclusion criterion.)

17 Laboratory testing (hematology, clinical chemistry, urinalysis, tumor markers, pregancy test) is performed according to the present standards in oncology. Accordingly, all parameters evaluated including the frequency of their evaluation are part of clinical routine and none of them is explicitly study-related.

18 QoL questionnaire EQ-5D-5L during screening within 21 days before randomisation, at every restaging visit as well as at the EoT visit.

19 Only required as optional translational research in patients with residual archival FFPE tumor tissue samples. Shipment of tissue tumor samples to Prof. Dr. med. Dominik Modest, Charité - Universitätsmedizin Berlin; shipment during screening and after start of study treatment permitted.

20 About 20 mL blood in Streck – cell-free DNA blood collection tubes, 10 mL EDTA blood and 10 mL serum blood have to be collected at each of the following time points: screening within 21 days before randomisation, at the first restaging and upon progression on study treatment or at the EoT visit (only if study treatment was discontinued permanently without occurrence of progression, e.g. due to unacceptable toxicity). As translational research optional.

21 Only documentation of concomitant medication administered within 28 days before randomisation and during study treatment until the EoT visit.

22 Documentation of second-line treatment and subsequent treatment lines as reported by the investigator: list of substances administered as second-line and in subsequent treatment lines (if applicable) including treatment duration (date of first and last administration), efficacy of second-line treatment and subsequent treatment lines
